# Chronic Inflammation in Obesity and the Metabolic Syndrome

**DOI:** 10.1155/2010/289645

**Published:** 2010-07-14

**Authors:** Rosário Monteiro, Isabel Azevedo

**Affiliations:** Department of Biochemistry (U38-FCT), Faculty of Medicine, University of Porto, Al. Prof. Hernâni Monteiro, 4200-319 Porto, Portugal

## Abstract

The increasing incidence of obesity and the metabolic syndrome is disturbing. The activation of inflammatory pathways, used normally as host defence, reminds the seriousness of this condition. There is probably more than one cause for activation of inflammation. Apparently, metabolic overload evokes stress reactions, such as oxidative, inflammatory, organelle and cell hypertrophy, generating vicious cycles. Adipocyte hypertrophy, through physical reasons, facilitates cell rupture, what will evoke an inflammatory reaction. Inability of adipose tissue development to engulf incoming fat leads to deposition in other organs, mainly in the liver, with consequences on insulin resistance. The oxidative stress which accompanies feeding, particularly when there is excessive ingestion of fat and/or other macronutrients without concomitant ingestion of antioxidant-rich foods/beverages, may contribute to inflammation attributed to obesity. Moreover, data on the interaction of microbiota with food and obesity brought new hypothesis for the obesity/fat diet relationship with inflammation. Beyond these, other phenomena, for instance psychological and/or circadian rhythm disturbances, may likewise contribute to oxidative/inflammatory status. The difficulty in the management of obesity/metabolic syndrome is linked to their multifactorial nature where environmental, genetic and psychosocial factors interact through complex networks.

## 1. Obesity and the Metabolic Syndrome

The incidence of the metabolic syndrome is high and increasing. Metabolic syndrome refers to a constellation of disturbances including glucose intolerance, central obesity, dyslipidemia (hypertriglyceridemia, elevated nonesterified fatty acids (NEFAs), and decreased high-density lipoprotein (HDL) cholesterol), and hypertension [1]. It can present in several forms, according to the combination of the different components of the syndrome, and it is well established that it increases the risk for the development of cardiovascular disease, type 2 diabetes, and cancer [2–4]. However, it is not yet clarified how it begins and how the different components are causally connected among them. Different study groups have drawn special attention to one or another component. For example, the American Association of Endocrinology does not consider obesity as a component and highlights the importance of insulin resistance to the syndrome [5]. As a matter of fact, obesity and the metabolic syndrome do not completely overlap, evidence being now clear as to the existence of “benign” obesity [6–10]. In this metabolically healthy obese phenotype plasma concentrations of adiponectin are high, in good agreement with the effect of adiponectin overexpression in ob/ob mice that results in expansion of fat mass and protection against metabolic comorbidities [7]. The initial definition of the World Health Organization considered insulin resistance a key feature of the metabolic syndrome [11, 12], while the more recent National Cholesterol Education Program (NECP):Adult Treatment Panel III (ATP III) definition adds equal weight to any of the components of the syndrome: glucose intolerance, obesity, hypertension, and dyslipidemia [13]. 

Welsh et al. investigated the causal direction of the relationship between adiposity and inflammation using a bidirectional Mendelian randomization approach and came to the conclusion that greater adiposity conferred by fat mass and obesity-associated gene and melanocortin receptor 4 single nucleotide polymorphisms led to higher C-reactive protein (CRP) levels, with no evidence for any reverse pathway [14]. Although this interesting finding needs to be confirmed and extended to other inflammatory markers, it supports the emphasis we are giving to adipose tissue in metabolic syndrome-associated chronic inflammation [15]. Whatever its origin, be it or not obesity the main initiator, the chronic low-grade inflammatory condition that accompanies the metabolic syndrome has been implicated as a major player in both the installation of the syndrome and its associated pathophysiological consequences [16]. In good agreement with this interpretation of things, weight loss of obese patients is repeatedly verified to be associated with a decrease of inflammation biomarkers [17–21] accompanied by improvement of metabolic parameters, namely, insulin sensitivity [22–26].

## 2. The Inflammatory Response

Inflammation is a physiological response of the organism to harmful stimuli, be they physical, chemical, or biological. The response provided usually conducts to the reestablishment of homeostasis. It involves the coordinated action of many cell types and mediators, whose intervention depends on the nature of the initial stimulus and ensuing responses thereafter. The normal acute inflammatory response involves the delivery of plasma components and leucocytes to the site of insult and is initiated by tissue-resident macrophages and mast cells leading to a production of different types of inflammatory mediators (chemokines, cytokines, vasoactive amines, eicosanoids, and products of proteolytic cascades) [16]. The activated endothelium allows extravasation of neutrophils and soluble components to the tissue, where they become activated releasing toxic agents and proteolytic enzymes to the extracellular milieu [27]. If successful, the injurious agent is eliminated and inflammation resolution and tissue repair follow. This is achieved by switching the lipid mediators from proinflammatory (e.g., prostaglandins) to anti-inflammatory and pro-resolution ones (lipoxins, resolvins, and protectins) and by the action of tissue-resident and newly recruited macrophages [27]. Tissue leucocytes undergo apoptosis and are phagocytised by the macrophages that leave the inflamed site by lymphatic drainage [28]. The apoptosis of inflammatory cells is a nonphlogistic physiological process of removing dead cells and is essential for the resolution of inflammation [16] with the added advantage that after engulfing these apoptotic cells, macrophages acquire a phenotype conductive to resolution, releasing anti-inflammatory signals such as interleukin-(IL-) 10 and transforming growth factor *β* (TGF*β*) [29]. However, if the neutralization and removal of the noxious stimuli or even if the clearance of apoptotic inflammatory cells from the inflamed tissue fail, the inflammatory process persists and a state of chronic inflammation or autoimmunity may arise with different agents being recruited, namely, T lymphocytes and the development of lymphoid infiltrates in the tissue [30]. It has recently been demonstrated that the chronic inflammatory condition associated with morbid obesity is characterized by a continuous activation of the innate immune system [31].

## 3. Inflammation in Obesity and the Metabolic Syndrome

The inflammatory state that accompanies the metabolic syndrome shows a quite peculiar presentation, as it is not accompanied by infection or sign of autoimmunity and no massive tissue injury seems to have taken place. Furthermore, the dimension of the inflammatory activation is not large and so it is often called “low-grade” chronic inflammation. Other researchers have attempted to name this inflammatory state as “metaflammation”, meaning metabolically triggered inflammation [32], or “parainflammation” as a term to define an intermediate state between basal and inflammatory states [33]. Whatever the term used, the inflammatory process that characterizes the metabolic syndrome seems to have its own unique features, its mechanisms being far from fully understood [15].

Recent studies have been confirming the positive association between obesity indices and inflammatory markers, mainly CRP protein in women [34, 35], but also other inflammatory markers, both in women and men [31, 36].

Increasing incidence of the metabolic syndrome all over the world accompanies adoption of the so-called modern Western lifestyle [37, 38]. The main negative features of this lifestyle include stress (long-term and continuous, psychological), positive energy balance (excessive energy intake and low physical activity), low-quality food (both high fat and energy dense, and at the same time poor in micronutrients), and disruption of chronobiology. An acute disturbance in any of the physiological regulatory systems evokes reactions that tend to reestablish equilibrium. When the stimuli, even of moderate magnitude, tend to be repetitive or chronic, change and allostasis in one system impact on the other, and vicious cycles are created and reinforced [1]. 

A state of positive energetic balance, with fat accrual along time, demands plasticity of the adipose tissue, which includes formation of new adipocytes and also physical compliance of the anatomical spaces for adipose tissue expansion [39]. Otherwise two deleterious phenomena ensue, hypertrophic growth of adipocytes that will rupture more and more frequently and fat deposition in organs other than adipose tissue [40], mainly in the liver, with local (nonalcoholic fatty liver disease) and systemic (insulin resistance) consequences. It is well known that the lack of adipose tissue, as evident in lipodystrophy [41], may equally lead to the development of metabolic syndrome. 

After a meal, fatty acids are taken up by adipocytes, and during fasting or increased expenditure periods, the fatty acids are released to the blood stream. This is usually achieved through the coordinated action of hormones, catecholamines and insulin being the chief regulators of this balance [42]. Not only is the adipocyte metabolism altered by these hormones, but these hormones also regulate blood flow into the adipose tissue through the regulation of vascular tone, such that there is increased blood supply when the organ is metabolically active [43]. Inadequate blood supply in a growing adipose tissue results in reduced oxygenation that may also contribute to inflammation [44]. 

The homeostatic capacity of adipose tissue development, with adipocyte hyperplasia responding to fat accommodation needs, has its limits and may contribute, in people that are not able to develop benign obesity, to adipocyte hypertrophy and the inflammatory response. Obesity researchers aimed, for some time, to find and study antiadipogenic agents to combat/prevent obesity, but that concept is no longer defensible [45–48].

### 3.1. Adipocyte Dysfunction and Inflammation

An increase in circulating concentration of NEFA [42] reflects the inability of the adipose tissue to buffer the excess nutrient intake and is related to the dyslipidemic state that is typical of the metabolic syndrome. When overload becomes present, the liver increases the production of apo-B containing particles that carry triacylglycerols to the adipose tissue resulting in low-density lipoprotein (LDL) formation [49]. This occurs in visceral adipose tissue very efficiently and this depot is also more capable of releasing lipids in times of requirement. The subcutaneous adipose tissue has usually a much larger capacity to store lipids given its usually larger size. This may explain why subcutaneous adipose tissue appears to be protective in terms of metabolic syndrome and why men, who for genetic and hormonal reasons possess a smaller subcutaneous adipose tissue compartment, achieve earlier the limit of that depot and overuse the visceral depot. When the capacity of both locations is overwhelmed, the conversion of very low-density lipoprotein (VLDL) or similar particles is delayed and hypertriglyceridemia originates [42]. Furthermore, other tissues are used for lipid accumulation (e.g., liver, muscle, pancreas, and heart) [40]. As these organs are not able to store lipids without harm to their functions, lipotoxicity may be the result culminating, in the case of muscle, liver, and pancreas, in insulin resistance. 

Although it has become evident that adipocytes are involved in innate immunity, only recently was the presence of toll-like receptors (TLRs) in these cells described. The two most widely studied TLRs are TLR2 and TLR4, which are activated by bacterial lipoproteins and by lipopolysaccharide (LPS), respectively [50]. The engagement of either receptor leads to translocation of nuclear factor *κ*B (NF*κ*B) to the nucleus. In addition to their early recognized role in immunity, a participation in the regulation of metabolism is also being attributed to TLRs [50]. It has been shown that these receptors may be activated by specific types of lipids. It had already been demonstrated that the fatty acid moiety of TLR ligands was essential for their activation [51] and this led to the investigation of its possible activation by different sorts of lipids. Thus, it was discovered that saturated fatty acids activate both TLR2 and TLR4 and, instead, unsaturated fatty acids inhibit TLR-mediated signalling and gene expression [16]. This was also demonstrated with diet-derived saturated fatty acids, which increased TLR-mediated expression of IL-6 and tumor necrosis factor *α* (TNF-*α*), whereas unsaturated fatty acids had no effect alone but inhibited the saturated fatty acid-induced increase in TNF-*α* expression [16]. TLR4 has been found in murine preadipocytes 3T3-L1 and both TLR2 and TLR4 have been shown to be present and functional in human subcutaneous adipocytes [16]. Activation of TLRs results in synthesis of proinflammatory factors such as TNF-*α*, IL-6, and chemokines [16]. Considering the implication of the increase in circulatory NEFA in adipose tissue dysfunction, it is very likely that the activation of TLRs takes place in hyperlipidemic states, resulting in amplified inflammation and contributing to the development or aggravation of the metabolic syndrome [50].

There is compelling evidence showing that exposure of adipocytes to several types of stressors (oxidative stress, inflammatory cytokines, and elevated concentrations of fatty acids) induces cellular responses mediated by cellular kinases, including mitogen-activated protein kinases (MAPK; p38MAPK, c-Jun N-terminal kinase (JNK) and extracellular signal-regulated kinase), inhibitor of NF*κ*B kinase (IKK) *β*, mammalian target of rapamycin (mTOR), and various conventional and atypical protein kinases C (PKC). Some of these kinases involved in stress-sensing are related to the impairment of insulin action through the stimulation of insulin receptor substrate (IRS) serine phosphorylation but also often activate targets related to the inflammatory response [32]. The three main kinases that have been related to this inactivation of IRS are JNK, IKK and PKC [16]. They exert powerful effects on proinflammatory gene expression, through activation of activator protein-1 (AP-1) complexes and NF*κ*B [52]. 

Metabolic dysfunction due to metabolic overload also seems to be mediated through IKK*β*. The reduction of IKK*β* expression partly protects mice from obesity-induced insulin resistance, and the inhibition of this kinase achieved by high doses of salicylates has been shown to improve insulin sensitivity in humans and other experimental models [16]. PKC has also been shown to constitute an important interface between metabolic deregulation, inflammation, and insulin resistance. This kinase (isoform *θ* in skeletal muscle and *δ* in the liver) can be activated by fatty acid metabolites that accumulate due to metabolic pathway burden such as fatty acyl CoA and diacylglycerol, leading to inhibitory serine phosphorylation of IRS and attenuation of insulin signalling [16]. Furthermore, PKC*θ* is known to activate IKK and might contribute to insulin resistance and amplification of inflammation. 

The metabolic stress to which adipose tissue is subjected in obesity, together with other already mentioned stresses, results in organelle dysfunction, particularly in mitochondria and the endoplasmic reticulum (ER). The ER is a cytosolic organelle that participates in the regulation of lipid, glucose, cholesterol, and protein metabolism, apart from being the site of triacylglycerol droplet formation. In the obese, the adipocyte may be especially challenged, given that it is required to secrete large amounts of substances and synthesise lipids. Under such conditions, ER function may be impaired leading to the accumulation of misfolded or unfolded proteins in its lumen. As a way to cope with it, the stressed endoplasmic reticulum engages the unfolded protein response (UPR). The UPR functions via signalling through three branches, denoted for the three stress-sensing proteins found in the ER membrane: PKR-like eukaryotic initiation factor 2a kinase (PERK), inositol-requiring enzyme-1 (IRE-1), and activating transcription factor-6 (ATF-6) [53]. Their activation attenuates the cellular workload (decreasing protein translation, clearance, and degradation of excess proteins from the ER lumen) and induces repair (induction of an antioxidant response and of chaperone transcription to assist with the unfolded proteins) and ER biogenesis, towards recovery and survival of the cell. However, if the ER stress is not relieved, the UPR may also induce cell death via apoptosis. JNK, after activation by IRE-1, is an important effector in this action and, apart from this role, it may lead to a variety of other downstream effects depending on the cellular context, such as cell survival, inflammation, and insulin resistance. The activation of inflammation by the UPR also depends upon the IKK-NF*κ*B pathway, also through IRE-1*α*, resulting in increased TNF-*α* and IL-6 production, further supporting its contribution to insulin resistance [16]. 

Obesity is associated with deregulated lipid and carbohydrate metabolism. An increase in either one of these substrates will also increase the demand on the mitochondria and the utilization of the electron transport chain [54]. As in metabolically active tissues undergoing increased demand, there is usually relative hypoxia, together with the increased need for nutrient oxidation. This generates unusual amounts of reactive oxygen species. Oxidative stress activates kinases like JNK, p38 MAPK, and IKK that may directly interfere with insulin signalling or indirectly via induction of NF*κ*B and increased cytokine production [16].

Furthermore, the enhanced flux through alternative metabolic pathways leads to the generation of other metabolites that may themselves be related to impairment of insulin function and inflammation. Examples include fatty acyl CoA, diacylglycerol, which activate PKC serine kinases, and ceramide, which can undergo phosphorylation to ceramide-1-phosphate and promote inflammation or originate secondary metabolites with the same effect. Ceramide may also activate protein phosphatase 2A, which inactivates PKB/Akt and attenuates insulin response, contributing to insulin resistance [55].

### 3.2. Visceral Obesity

Adipose tissue pathogenicity differs according to adipose tissue localization, visceral, or subcutaneous [56]. Visceral adiposity seems to be an independent predictor of insulin sensitivity [57–59], impaired glucose tolerance [60], elevated blood pressure [61, 62], and dyslipidemia [58, 63]. Visceral fat is a highly active tissue from the metabolic point of view. It is apparently more susceptible to lipolysis than subcutaneous adipose tissue [64] and is associated with higher production of TNF-*α* [64, 65], plasminogen activator inhibitor-1 (PAI-1) [66], IL-6, and CRP [67]. On the other hand, it is a feabler producer of adiponectin, an adipokine more strongly correlated with subcutaneous fat [68]. Bahceci and collaborators [69] found a positive correlation between adipocyte size and TNF-*α*, IL-6, and high-sensitivity CRP. On the other hand, adiponectin was found to be negatively correlated with adipocyte size. 

Although visceral obesity seems to play a central role in the metabolic syndrome, being generally considered much more proinflammatory [70], not all patients with the syndrome present this feature. Perhaps more important than the amount of accumulated fat in the abdominal cavity is the size of abdominal adipocytes. We have shown that big adipocytes are more prone to rupture [45], and cell rupture will obviously constitute a focus of inflammation. Macrophage crowns surrounding dead adipocytes in adipose tissue, as shown by Cinti and collaborators [71], are compatible with this hypothesis.

As already stated, adipocytes behave as immune cells [72–75] and are able to synthesize and release a huge amount of proinflammatory adipokines and cytokines including leptin, resistin, PAI-1, IL-6, TNF*α*, retinol-binding protein 4 (see [16]), IL-1*β*, monocyte chemoattractant protein-1 (MCP-1), CRP, macrophage migration inhibitory factor (MIF) (see [76]), chemokines from the CC and CXC families [77], and more recently described cytokines such as IL-18 [78] and IL-33 [79], most of which, if not all, are involved in insulin resistance [76, 80]. But beyond the capacity of adipocytes to secrete these and possibly more proinflammatory cytokines, under circumstances of obesity and/or nutrient overload, adipose tissue macrophages do also provide their counterpart of insulin-resistance inducing cytokines. Moreover, obese adipose tissue do also contain lymphocytes that participate in and reinforce the inflammatory reaction and consequent insulin resistance [77, 81, 82].

Whereas subcutaneous adipocytes can also become hypertrophic, and most probably do also rupture, visceral adipocytes, besides being supported by much less dense connective tissue in comparison with subcutaneous adipose tissue, are frequently subject to sudden pressure variations associated with cough, physical exercises [83], and sleep apnea [84, 85]. Intra-abdominal pressure is higher in obese patients [86], what may also impact on adipocyte stability. Preadipocytes of upper-body obese women exhibit reduced differentiation and are more prone to apoptosis than preadipocytes isolated from adipose tissue of lower-body obese or lean women [87]. The factors involved in this preadipocyte behaviour deserve investigation [88]. Moreover, it has been proposed that visceral adipose tissue growth is mainly due to hypertrophy, while in other locations there may be mainly growth through hyperplasia [89]. The physical constrains presented inside the abdominal cavity may halt adipogenic differentiation of adipose tissue precursors reducing the number of competent cells to accumulate excessive energy ingested. As a matter of fact, it has been shown that stretching inhibits adipocyte differentiation [16].

In an interesting model of diet-induced metabolic syndrome, the fructose-fed rat, the animals exhibit a series of metabolic syndrome features but do not weigh more than controls. However, their abdominal adipocytes are hypertrophic, a modification prevented by blockade of the renin-angiotensin system [90]. Functional relationships between adipocytes and the renin-angiotensin-aldosterone system [91] or between adipocytes and adrenocortex/aldosterone [92] do probably deserve more attention. A critical role for the balance between gluco- and mineralocorticoid action in determining adipocyte responses implicated in obesity-associated inflammation and cardiovascular complications has recently been demonstrated [93].

Another characteristic of abdominal adipose tissue is its high metabolic activity and dense vascularisation. This high vascularisation is most probably due to the action of angiogenic/proinflammatory factors, of which leptin constitutes an example [94, 95]. On the other hand, increased intra-abdominal pressure, as well as pressure variations, may easily create periods/zones of hypoxia, contributing to the production of hypoxia inducible factors. Vascular endothelial growth factor (VEGF), leptin, adenosine, and substance P, among others, will impact on other features of the metabolic syndrome. Furthermore, hypoxia by itself inactivates the adiponectin promoter [96].

### 3.3. Diet, Microbiota, and Inflammation

Among the complex interaction between genetic, metabolic, and environmental factors that is most probably associated with the present prevalence of obesity and metabolic syndrome, dietary patterns are considered of central importance. In these, attention has been focused over calories, amounts, and proportions of macronutrients, and their effects on the energetic balance by themselves, and through metabolic regulators. Only recently have the acute effects of food ingestion, taking into consideration the type of food, and the specific effects of some nutrients, namely, fatty acids, began to be studied in relation with obesity and inflammation.

Total dietary fat and saturated fat are associated with insulin resistance and high blood pressure as well as obesity-related inflammation [97]. An immediate postprandial increase in plasma inflammatory markers after a high-fat meal had been shown in abdominally obese men [98]. Consumption of a saturated fatty acid-rich diet resulted in a proinflammatory “obesity-linked” gene expression profile, whereas consumption of a monounsaturated fatty acid-rich diet caused a more anti-inflammatory profile [99]. Moreover, acute dietary n-3 PUFA dietary supplementation has been shown to improve fasting as well as postprandial lipid metabolism and components of the associated inflammatory response in the JCR:LA-cp rat [100]. Increased central and overall adiposity in children is associated with higher circulating adipocyte protein 2 (aP2) concentrations, high dietary intakes of total fat and saturated fat influencing aP2 association with insulin resistance and inflammation [101]. Intakes of total fat and antioxidant vitamins have been shown to be determinants of subclinical inflammation in the same children [102].

It must, however, be taken into account that inflammatory markers increase even after a mixed meal, more so in children with central obesity [103]. The proinflammatory effect of a high-fat diet per se is shown in a most interesting investigation by the group of Rankin: people put under a weight loss diet and that effectively lost weight increased the concentration of an inflammation marker when the diet was high-fat, differently from a low-fat high carbohydrate diet where the inflammatory marker decreased with the loss of weight. They also showed that the proinflammatory effect of the high-fat diet was not dependent on oxidative stress [104, 105]. These results are corroborated in a recently published experimental research work in mice, where it has been shown that a shift from a high-fat to a high-carbohydrate diet improved levels of adipokines and proinflammatory cytokines [106].

Intestinal microbiota, microorganisms which are known to contribute to digestion and metabolism, have barely been taken into consideration in this discussion, but for some recent papers [107–111]. However, the extension and metabolic gene *repertoire* of our microbiota are compatible with huge metabolic consequences for the host depending on the microbiota constitution.

Comparison of microbiota from obese and nonobese subjects has shown different proportions of certain species [112], but it remains unknown what is cause and consequence, or the metabolic consequences, for the host, of harboring different microbiota. This remains an almost virgin soil, in spite of the putative importance of conditioning the evolution of the microbiota in the metabolic interest of the host. Some associations between dietary patterns and resistance to obesity, namely, the importance of the diet acid load [113], nondigestible plant material [114], or the richness in certain minerals [115] may indeed include effects of those elements on the microbiota. 

Cani and collaborators have demonstrated that high-fat feeding increases intestinal permeability and the plasma concentration of LPS, that is, metabolic endotoxemia, and that changes in gut microbiota control this metabolic endotoxemia and inflammation [116]. Therefore, high-fat feeding may have direct proinflammatory /anti-inflammatory effects, depending on the nature of fatty acids, long-term inflammatory consequences related with overload of adipose tissue, and indirectly modulate metabolic endotoxemia and inflammation through effects on the microbiota.

## 4. Conclusion

**Figure 1:**
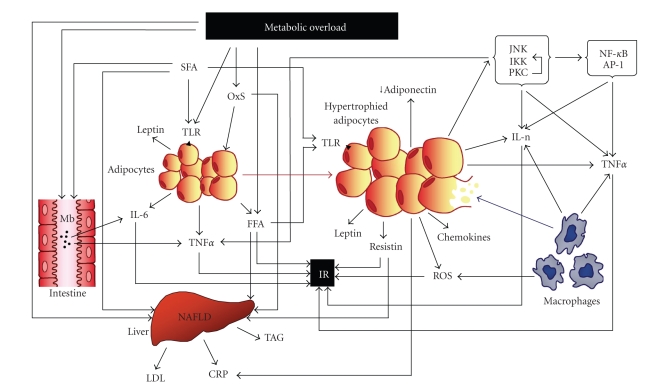
Overview of the complex interplay between obesity-inflammation-metabolic syndrome: metabolic overload impacts on adipose tissue, leading to organelle stress with production of ROS and adipokines, as well as activation of kinases that potentiate the transcription of inflammatory genes and interfere with insulin signaling. Hyperthrophy facilitates rupture of adipocytes which attract and activate macrophages that markedly reinforce the inflammatory process through further production of ROS and inflammatory cytokines. Production of adiponectin, an anti-inflammatory cytokine, is reduced. Increase of FFA concentration, namely, SFA, coming both from feeding and adipose tissue overflow, accumulates in the liver, among other organs. Fat accumulation in the liver leads to overproduction of LDLs and, together with IL-6, of CRP. NAFLD is a frequent consequence of these metabolic dysregulations, and all this impacts on insulin sensitivity. SFA activates TOLL-like receptors in adipocytes, contributing to the activation of the inflammatory response. Fat has also effects on intestinal permeability and on the microbiota, with systemic inflammatory consequences. Most excess metabolites and cytokines produced throughout these processes converge on insulin resistance, a central characteristic of the metabolic syndrome. AP-1: activator protein-1; CRP: C-reactive protein; FFA: free (nonesterified) fatty acids; IL-n: interleukins; IKK: inhibitor of NF-*κ*B kinase; IL-6: interleukin-6; Int: intestine; IR: insulin resistance; JNK: c-Jun N-terminal kinase; LDL: low density lipoprotein; M: microbiota; NAFLD: nonalcoholic fatty liver disease; NF-*κ*B: nuclear factor *κ*B; OxS: oxidative stress; ROS: reactive oxygen species; PKC: protein kinase C; SFA: saturated fatty acids; TAG: triacylglycerols; TLR: TOLL-like receptors; TNFalpha: tumour necrosis factor alpha.

It has become evident that the inflammatory condition that is associated with obesity and overweight plays an important part in the aetiology of the metabolic syndrome and largely contributes to the related pathological outcomes. From the reasons here presented, it seems likely that adipocyte dysfunction lies at the bottom of this question, its homeostatic functions being overwhelmed due to metabolic overload. From then on, several vicious cycles exacerbate the disturbances and lead to an inflammatory response, most intense when adipocytes reach large dimensions and easily rupture for mechanical reasons. In parallel with this, the effects of high-fat diets, frequently consumed by obese subjects, add to the inflammatory fire, both directly, when the fat is rich in saturated fatty acids, and indirectly, through effects on the microbiota and intestinal permeability (Figure 1). 

Progress in the knowledge of this highly complex network of pathophysiological events that include multiple cell types, cytokines, nutrients, in a variable nervous and hormonal status, as well as specific physical constraints and microbial colonization, will open the way for effective therapeutic interventions. Inflammatory mediators are already showing their usefulness as biomarkers of the metabolic/inflammatory/disease-prone status of patients with the metabolic syndrome.

But diet will remain playing a crucial role in multiple aspects of this large picture. And, although much remains to be known in what respects nutrition, the biggest challenge will be to reinstall a nonobesogenic lifestyle.

## List of Abbreviations

AP-1: Activator protein-1
aP2: Adipocyte protein 2
ATF-6: Activating transcription factor-6
ATP III: Adult treatment panel III
CoA: Coenzyme A
CRP: C-reactive protein
ER: Endoplasmic reticulum
HDL: High-density lipoprotein
IKK: Inhibitor of nuclear factor *κ*B kinase
IL: Interleukin
IRE-1: Inositol-requiring enzyme-1
IRS: Insulin receptor substrate
JNK: C-Jun N-terminal kinase
LDL: Low-density lipoprotein
LPS: Lipopolysaccharide
MAPK: Mitogen-activated protein kinase
MCP-1: Monocyte chemoattractant protein-1
MIF: Migration inhibitory factor
mTOR: Mammalian target of rapamycin
NECP: National cholesterol education program
NEFA: Nonesterified fatty acids
NF*κ*B: Nuclear factor *κ*B
PAI-1: Plasminogen activator inhibitor-1
PERK: PKR-like eukaryotic initiation factor 2a kinase
PKB: Protein kinase B
PKC: Protein kinase C
TGF*β*: Transforming growth factor *β*
TLR: Toll-like receptor
TNF: Tumor necrosis factor
UPR: Unfolded protein response
VEGF: Vascular endothelial growth factor
VLDL: Very low-density lipoprotein.
